# Ferromagnetic Half-Metal Cyanamides Cr(NCN)_2_ Predicted from First Principles Investigation

**DOI:** 10.3390/ma13081805

**Published:** 2020-04-11

**Authors:** Zhilue Wang, Shoujiang Qu, Hongping Xiang, Zhangzhen He, Jun Shen

**Affiliations:** 1 School of Materials Science and Engineering, Tongji University, 4800 Caoan Road, Shanghai 201804, China; wangzl@tongji.edu.cn (Z.W.); qushoujiang@tongji.edu.cn (S.Q.); junshen@tongji.edu.cn (J.S.); 2 State Key Laboratory of Structural Chemistry, Fujian Institute of Research on the Structure of Matter, Chinese Academy of Sciences, Fuzhou 350002, China

**Keywords:** first principles theory, transition metal compounds, magnetism

## Abstract

The stability, physical properties, and electronic structures of Cr(NCN)_2_ were studied using density functional theory with explicit electronic correlation (GGA+*U*). The calculated results indicate that Cr(NCN)_2_ is a ferromagnetic and half-metal, both thermodynamically and elastically stable. A comparative study on the electronic structures of Cr(NCN)_2_ and CrO_2_ shows that the Cr atoms in both compounds are in one crystallographically equivalent site, with an ideal 4+ valence state. In CrO_2_, the Cr atoms at the corner and center sites have different magnetic moments and orbital occupancies, moreover, there is a large difference between the intra- (12.1 meV) and inter-chain (31.2 meV) magnetic couplings, which is significantly weakened by C atoms in Cr(NCN)_2_.

## 1. Introduction

Recently, high-valence chromium oxides have attracted much attention because of their unusual physical properties and complicated microscopic mechanism [1,2,3,4,5,6,7,8]. For example, CaCrO_3_ as a rare metallic antiferromagnet was reported to have a Bose–Einstein condensate at *T_N_* [2,5]. The quasi-one-dimensional Hollandite-type structure K_2_Cr_8_O_16_ undergoes an unconventional metal–insulator transition while maintaining the ferromagnetic state [3,8]. CrO_2_, as a simplest Cr^4+^ compound, is a half-metal ferromagnet (T_C_ = 390 K) and has been widely used in magnetic recording media. It has been widely studied both in experiment and theory; however, the origin of its half-metal ferromagnetism remains controversial and unclear. Early theoretical studies suggest a self-doping double exchange mechanism for describing the material’s intertwined metallicity and ferromagnetism [9,10]. Shim et al. indeed observed two different Cr ions coexistence in CrO_2_ using ^53^Cr nuclear magnetic resonance (NMR), supporting the self-doping and double exchange mechanism [11]. Latterly, Takeda et al. also observed the presence of two Cr sites using the orbital-resolved NMR method, however, they revealed that two Cr ions at the corner and body center sites have the same valence state, but different orbital occupancies [12]. A local orbital order takes place with breaking of the local symmetry. They ascribed it to the negative charge transfer between chromium and oxygen ions. Nevertheless, a realistic low-energy model derived from the first principles calculations presents that the direct exchange interactions and the magnetic polarization of the oxygen 2*p* band play a very important role in the stability of the ferromagnetic ground state of CrO_2_ instead of double exchange [13,14,15]. Thus, a clear and uniform microscopic model is desired to elucidate it.

Here, we predicted a new Cr^4+^-based compound Cr(NCN)_2_ by first principles theory. It has a tetragonal structure with a space group of *P*4_2_/*mnm*, similar t-o CrO_2_. NCN^2−^as a pseudochalcogen umligand, has the same oxidation state as O^2−^, but different structure and electronegative. Experimental reports indicate that NCN^2−^-based 3*d* transition-metal compounds display rich physical and chemical properties, showing the similarity and difference to corresponding oxides [16,17,18,19]. For instance, Cr_2_NCN_3_ has the same crystal structure as Cr_2_O_3_ with the *R*–3*c* space group; however, it is a rare ferromagnetic semiconductor, quite different from antiferromagnetic Cr_2_O_3_ [17].

As in CrO_2_, Cr atoms in Cr(NCN)_2_ are octahedrally coordinated by nitrogen, forming edge-sharing octahedral ribbons along the *c* axis. However, the octahedra on adjacent ribbons are connected by NCN^2−^, leading to a larger distance of 5.906 Å, not like those in CrO_2_ that share an apex O^2−^ with a shorter distance of 3.450 Å. The large inter-chain distance induces a weak magnetic interaction, and as a result Cr(NCN)_2_, provides a simpler theoretical model for disclosing the mechanism behind the ferromagnetic and half-metal property for CrO_2_, even for other Cr^4+^-based magnetic compounds. Therefore, in this paper, besides predicting the crystal structure, we also calculated the electronic and magnetic structure through the use of density functional theory with explicit electronic correlation (GGA+*U*). In our study, both GGA and GGA+*U* calculations present a ferromagnetic half-metal for Cr(NCN)_2_. The electronic structures show the Cr atoms in both Cr(NCN)_2_ and CrO_2_ are in one crystallographically equivalent site, with an ideal 4+ valence state. In CrO_2_, the Cr atoms at the corner and center sites have the different magnetic moments and orbital occupancies, moreover, there is a large difference between the intra- (12.1 meV) and inter-chain (31.2 meV) magnetic couplings, which is significantly weakened by C atoms in Cr(NCN)_2_.

## 2. Methods

As an AB_2_-type compound, two kinds of initial crystal structures were chosen for Cr(NCN)_2_, that is, tetragonal *P*4_2_/*mnm* from CrO_2_ [20] and orthorhombic Pnnm from the high-pressure CrO_2_ and M(NCNH)_2_ (M = Fe, Co, Ni) [21,22]. This is because, as an analog of O^2−^ ligand, NCN^2−^-based 3*d* transition metal compounds have been proven to own a similar crystal structure as corresponding oxides [16,17,18,19]. The initial structures were optimized by letting all lattice parameters and the positions of Cr, C, and N relax simultaneously until self-consistency was achieved.

The calculations were based on density functional theory [22,23] in which the ground state properties of a many-electron system are determined by an electron density with three spatial coordinates instead of *N* electrons with 3*N* spatial coordinates many-body problem, and performed using the plane–wave pseudopotential Vienna ab initio Simulation Package [24,25,26], which is a well-tested code and has been successfully used to calculate a great variety of materials [17,18,19,27,28,29,30]. The generalized gradient approximation [31] was used for the exchange-correlation functional, which was formulated by Perdew, Burke, and Ernzerhof (GGA-PBE). The projector-augmented wave (PAW) method was employed with a cutoff energy of 800 eV, which was proposed by Blöchl [32] and implemented by Kresse and Joubert [33]. A uniform mesh grid with an actual spacing of 0.031 Å^−1^ was used to sample the complete Brillouin zone (*k*-point grid of 4 × 4 × 10). Brillouin zone integrations were performed with the Methfessel–Paxton method for structure optimization and the tetrahedron method with Blöchl’s correction for electronic structure [34]. The PAW pseudopotentials are 2*p*^6^3*d*^5^4*s*^1^ for Cr, 2*s*^2^2*p*^2^ for C, 2*s*^2^2*p*^3^ for N, and 2*s*^2^2*p*^4^ for O. Electron–electron Coulomb interactions in combination with the self-interaction correction were considered for the Cr atom in the rotationally invariant method (GGA+*U*) with an effective Hubbard parameter *U*_eff_ = *U* − *J* [35,36], which is set as 3.0 eV, obtained from the previous theoretical studies on the oxides based on Cr^4+^ [6,37].

Density-functional perturbation theory (DFPT) [38] in Vienna ab initio Simulation Package combined with the analysis program PHONOPY [39] was used to calculate the phonon frequencies of Cr(NCN)_2_. The 2 × 2 × 2 supercell including 112 atoms was used to calculate the force constants. A total of 101 *k*-points was used to sample each segment of band paths in order to obtain phonon dispersion relations.

## 3. Results and Discussion

**Figure 1:**
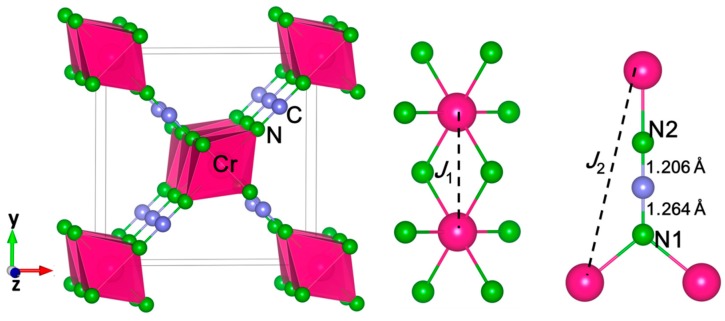
Crystal structures of Cr(NCN)_2_ (left) and the coordination environments of the Cr^4+^ ion (middle) and the NCN^2–^ ions (right). The Cr, N, and C are in pink, green, and gray, respectively.

The calculated results show that both initial structures (*P*4_2_/*mnm* and *Pnnm*) are stable in the same ground state, that is, *P*4_2_/*mnm*. Thus, Cr(NCN)_2_ was predicted to be a tetragonal crystal with the same space group as CrO_2_ in ambient condition (Figure 1), and Table S1 in the Supporting Information shows all of the theoretical structure parameters. This is quite similar as the scenario in Cr^3+^ ion-based compound Cr_2_(NCN)_3_, which has the same crystal structure as Cr_2_O_3_. The calculated lattice parameters are *a* = 8.04 Å, *c* = 3.1318 Å, *V* = 202.445 Å, and *Z* = 2. The Cr^4+^ ion in the Wyckoff position 2*a* (0 0 0) is coordinated by six nitrogen atoms, leading to a slightly flattened octahedral coordination with four Cr–N bonds at 2.023 Å and two shorter Cr–N bonds at 1.934 Å. The average Cr–N bond length is 1.993 Å, close to the sum of Cr^4+^ and N^3−^ effective ionic radii [40] of 2.01 Å. Edge-sharing CrN_6_ octahedra form a single chain along the *z*-axis with a Cr–Cr distance of 3.132 Å (Figure 1). C atoms is in Wyckoff position 4 g (0.2238 0.2238 0) and N atoms occupy in two different positions, that is, N1 4 g (0.1126 0.1126 0) and N2 4 g (0.3299 0.3299 0), forming a strictly linear [N-C-N]^2−^ unit (∠N-C-N = 180°). The bond lengths between C and N are 1.264 Å (C-N1) and 1.206 Å (C-N2), indicating the asymmetrical structure of NCN^2−^. The theoretical predicted Cr(NCN)_2_ is different from other symmetrical 3*d* transition-metal carbondiimides MNCN (M = Mn, Fe, Co, Ni) and Cr_2_NCN_3_, in which two C–N bonds are the same, around 1.23 Å. This asymmetric structure results from the coordination of N atoms, N1 is connected by two Cr atoms and one C, but N2 is connected by one Cr and one C. Until now, this asymmetrical structure has only been reported in *d*^10^ nonmagnetic cyanamides Ag_2_NCN [41], HgNCN (II) [42], CdNCN (*R*3*m*) [43], and PbNCN [44]. For example, in Ag_2_NCN, there is a single bond C–N 1.270 Å and a triple bond C≡N 1.187 Å. In Cr(NCN_)2_, the edge-sharing CrN_6_ chains are connected by the asymmetrical [N–C≡N]^2–^, as a result of a large distance between CrN_6_ chains of about 5.906 Å. Therefore, Cr(NCN)_2_ would be the first transition-metal cyanamide with partially filled 3*d* orbitals.

**Figure 2:**
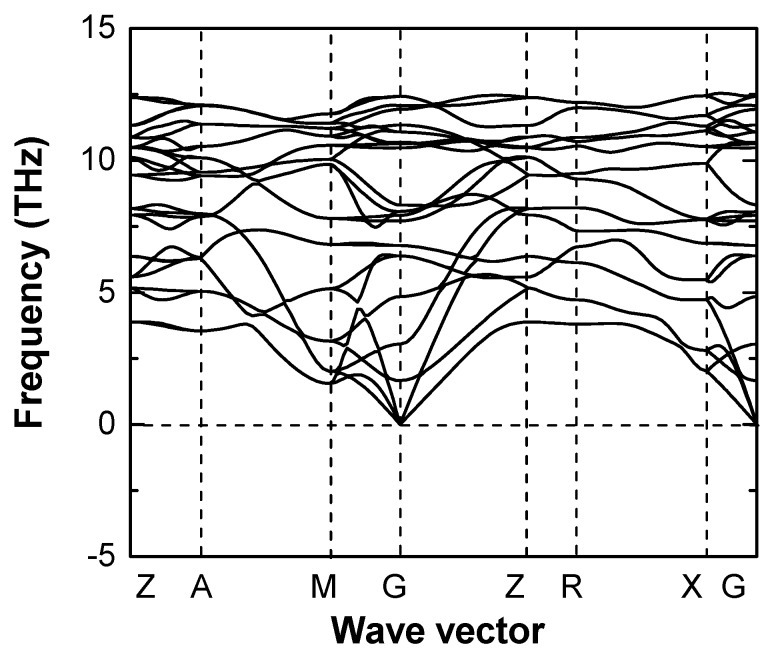
Calculated phonon dispersion relations of Cr(NCN)_2_ in ferromagnetic state. Imaginary phonon frequencies are shown by negative values.

The possible reaction routes were summarized in Equations (S1) and (S2) (see Supporting Information), based on successfully synthesized compounds Cr_2_NCN_3_ and MNCN (M = Mn, Fe, Co, Ni) [16,17]. The calculated reaction energies are −7.46 eV and −4.74 eV, respectively, indicating that the proposed reactions are possible candidates. The formation enthalpy, which is calculated from the direct reaction (Equation (S3)) with elements, is −13.54 eV, suggesting that Cr(NCN)_2_ is thermodynamically stable relative to the elements. Figure 2 shows the phonon dispersion curves of Cr(NCN)_2_ in the ferromagnetism, which is the most stable magnetic state and will be discussed below. It has no imaginary modes and hence is dynamically stable.

To achieve quantum-chemical insight into the conductivity and magnetic properties, we performed electronic-structure calculations for Cr(NCN)_2_ and CrO_2_ in *P*4_2_/*mnm* structure. Considering the structure as shown in Figure 1, two effective magnetic interactions were expected for Cr(NCN)_2_, the intra-chain *J*_1_ (two neighbors) and inter-chain *J*_2_ (eight neighbors) between the “nearest-neighbor” Cr ions as corresponding to the Cr–N–Cr along CrN_6_ octahedral chain and Cr–N–C≡N–Cr superexchange path between chains, respectively (see Figure 1). Thus, we considered a ferromagnetic (FM) and two anti-ferromagnetic structures (AFM1 and AFM2), shown in Figure S1 in the Supporting Information, needed to identify the most probable ground state magnetic structure and estimate the magnitudes of *J*_1_ and *J*_2_. As described in CuNCN [18,19], based on the Heisenberg spin Hamiltonian,

(1) $$\hat{H}=-\underset{i<j}{\sum }J_{ij}{\hat{S}}_{i}\cdot {\hat{S}}_{j}$$

the total energies per unit cell (two formula units) of FM, AFM1, and AFM2 can be written as

(2) $$E_{FM}=\left(-2J_{1}-8J_{2}\right)\left(N^{2}/4\right)$$

(3) $$E_{AFM1}=\left(-2J_{1}+8J_{2}\right)\left(N^{2}/4\right)$$

(4) $$E_{AFM2}=2J_{1}\left(N^{2}/4\right)$$

where *N* is the number of unpaired electrons, *N* = 2 for Cr^4+^ ion. Thus, we can extract *J*_1_ and *J*_2_ from

(5) $$J_{2}=\left(E_{AFM1}-E_{FM}\right)/\left(4N^{2}\right)$$

(6) $$J_{1}=\left(E_{AFM2}-E_{FM}\right)/\left(N^{2}\right)-2J_{2}$$

The total energies (Table 1) as calculated from GGA and GGA+*U* evidence that a ferromagnetic structure is most stable for both Cr(NCN)_2_ and CrO_2_, with the result of CrO_2_ consistent with previous experimental and theoretical reports [12,13]. The effective exchange parameters of *J*_1_ and *J*_2_ listed in Table 1 were estimated from the total energies. In the GGA context, the calculated total saturation magnetic moment *M*_tot_ is 2.01 *μ*_B_ f. u. and the spin saturation moment for Cr^4+^ ion *M*_Cr_ is 2.18 *μ*_B_ in Cr(NCN)_2_, larger than the *S* = 1 scenario. A small negative spin moment about −0.06 *μ*_B_ was found for N^3−^. When including the Coulomb interaction (GGA+*U*), the *M*_tot_ increases to 2.26 *μ*_B_ f. u., and there present two Cr spin moments of 2.63 *μ*_B_ and 2.62 *μ*_B_ at the corner Cr1 (0 0 0) and body-center Cr2 (0.5 0.5 0.5) sites, respectively, with a small difference about 0.01 *μ*_B_. Accordingly, the spin moments of N^3−^ diverge to four different values, with an average of −0.11 *μ*_B_. The similar values of *J*_1_ and *J*_2_ calculated from the GGA+*U* calculation suggest that the couplings of the intra- and inter-chain are comparative.

For CrO_2_, GGA and GGA+*U* both present different spin saturation moments for Cr1 and Cr2, that is, 2.11 *μ*_B_ and 2.05 *μ*_B_ (GGA), and 2.38 and 2.31 (GGA+*U*), respectively, with the difference of 0.06 and 0.07 *μ*_B_, respectively. The apex and basal plane oxygens of the CrO_6_ octahedron also have different spin moments of −0.05 *μ*_B_ and −0.06 *μ*_B_ for GGA, and −0.13 *μ*_B_ and −0.12 *μ*_B_ for GGA+*U*, respectively. The total saturation magnetic moment increases to 2.09 *μ*_B_ in GGA+*U* from 1.97 in GGA. Obviously, Cr1 and Cr2 in CrO_2_ are nonequivalent in magnetism in spite of one crystallographic Cr site, nevertheless, this nonequivalence is not obvious in iso-structure Cr(NCN)_2_. The values of *J*_1_ and *J*_2_ calculated from the GGA+*U* calculation are 12.1 and 31.2 meV, respectively. This indicates that the coupling of the inter-chain is stronger than that of the intra-chain.

The scenario of two Cr sites (corner and center sites) with different spontaneous moments has been reported for CrO_2_ in experiments [9,10,11]. However, two different microscopic mechanisms are proposed: (1) a mixed valence state of Cr^+4±δ^ resulting from a self-doping effect activates the double exchange mechanism, and thus induces the metallic ferromagnetism [9,10,11]; (2) the two Cr sites do not have different valence states, but have 3*d* orbital occupation numbers different from each other owing to the negative charge transfer between chromium and oxygen ions; as a result, a local orbital order takes place with breaking of the local symmetry, leading to the difference between the body-center and corner Cr sites [12]. In our calculation, Cr1 and Cr2 in both CrO_2_ and Cr(NCN)_2_ are in one crystallographically equivalent site, with ideal 4+ valence states. Clearly, the nonequivalent magnetic sites do not result from the mixed valence states. In order to disclose the physical mechanism, we perform electronic-structure calculations in the following.

**Figure 3:**
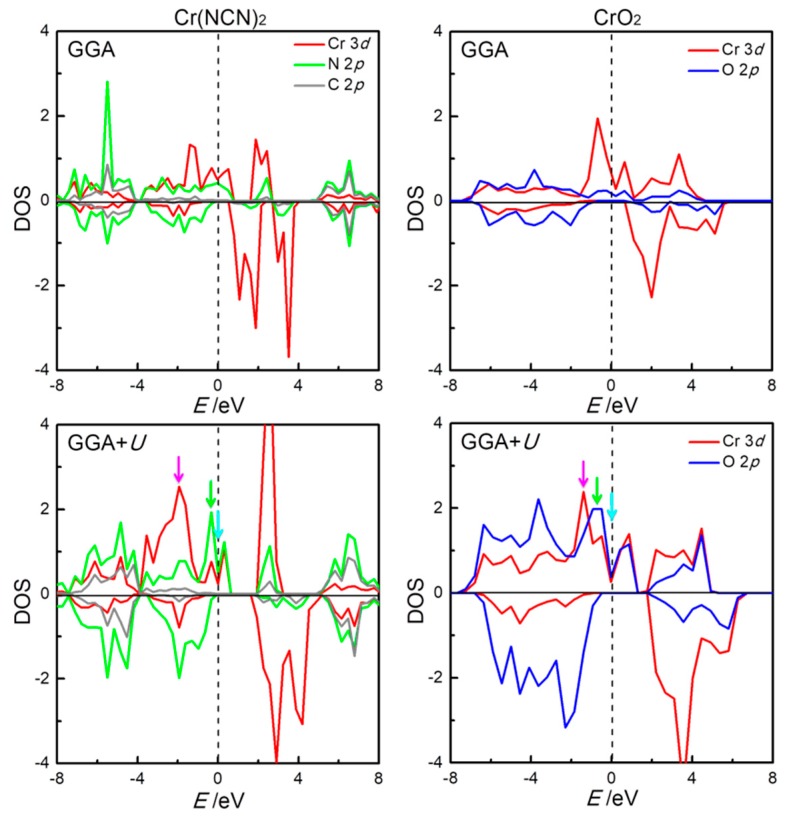
The density-of-states (DOS) of Cr(NCN)_2_ (left) and CrO_2_ (right) projected to Cr 3*d* (red), N 2*p* (green), C 2*p* (grey), and O 2*p* (blue) orbitals on the basis of GGA (upper row) and density functional theory with explicit electronic correlation (GGA+*U*) (lower row) calculations. The energy zero indicates the Fermi energy level.

The local density-of-states (DOS) within the FM states of Cr(NCN)_2_ and CrO_2_, as derived from GGA and GGA+*U* theory, are shown in Figure 3, with the band structure shown in Figure S2 in the Supporting Information. In the GGA description for Cr(NCN)_2_, there is a finite DOS on the Fermi level in majority spin with a strong Cr–N orbital mixing, while for minority spin, there is an energy gap of 0.62 *e*V between the highest occupied valence bands with N 2*p* character and the lowest-lying conduction bands of Cr 3*d* character, a *p*–*d* charge transfer insulating property. These suggest that Cr(NCN)_2_ is a half-metal compound, with 100% spin polarized thermally induced current at the Fermi level. Upon including an on-site Coulomb interaction (GGA+*U*), Cr(NCN)_2_ keeps the half-metallic character, with the *p*–*d* charge transfer energy gap increasing to 1.348 *e*V for the minority spin.

For CrO_2_, a half-metallic character is throughout for GGA and GGA+*U* calculations (Figure 3), in good agreement with experimental reports [12] and previous theoretical study [13]. It is a metallic property for majority spin with a strong Cr–O orbital mixing. For minority spin, the energy gaps of 1.348 and 2.254 *e*V from GGA and GGA+*U* theory are found between the highest occupied valence bands with O 2*p* character and the lowest-lying conduction bands of Cr 3*d* character, as a *p*–*d* charge transfer insulating property. In short, the electronic structures of Cr(NCN)_2_ from both GGA and GGA+*U* are similar to those of CrO_2_, both of which display the *p*–*d* charge transfer half-metal property.

**Figure 4:**
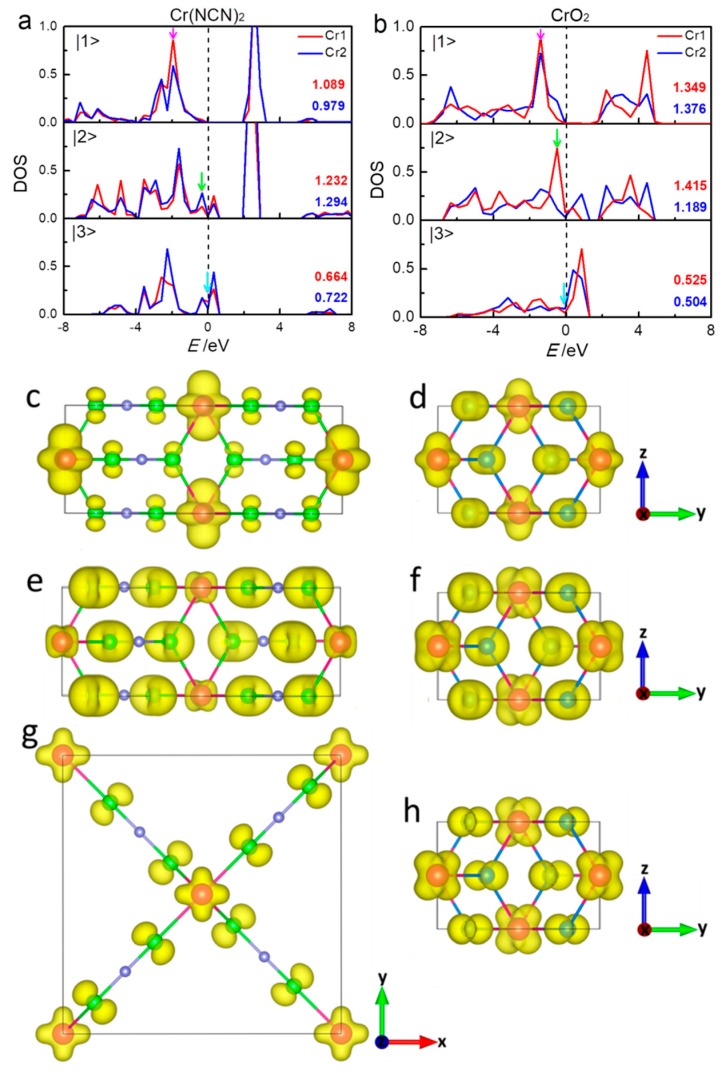
The partial densities-of-states in the spin majority of the Cr 3*d* orbitals on the basis of GGA+*U* calculations for Cr(NCN)_2_ (**a**) and CrO_2_ (**b**). The values inserted are the populations of the five Cr 3*d* orbitals in the majority channels, as calculated by integration (up to Fermi level *E*_F_) of the partial densities-of-states. The energy zero indicates the Fermi energy level. The corresponding three-dimensional electron density contour plots (e/Å^−3^) in the regions of (–2.20)–(–1.60) eV (**c**), (–0.69)–(–0.40) eV (**e**), and (–0.10)–0.0 eV (**g**) for Cr(NCN)_2_ and (–1.57)–(–1.35) eV (**d**), (–0.64)–(–0.37) eV (**f**), and (–0.10)–0.0 (**h**) eV for CrO_2_ (energy regions shown in blue, green, and purple arrows in Figure 3 and Figure 4a,b).

To better understand what is going on with the 3*d* orbitals of Cr1 (0.5 0.5 0.5) and Cr2 (0 0 0) ions in Cr(NCN)_2_ and CrO_2_, we consider the transformation of the corresponding orbital-projected DOS in more detail. In both compounds, the distortion of CrN_6_/CrO_6_ results in a symmetry lowering *O*_h_ to *D*_2*h*_, that is, a contraction of the octahedron along one of its threefold axes. Therefore, the forms of three *t*_2g_ orbitals in the global coordinate frame are $|1\rangle =\pm \frac{1}{2}|xy\rangle +\frac{\sqrt{3}}{2}|3z^{2}-r^{2}\rangle$, $|2\rangle =\frac{1}{\sqrt{2}}|yz\rangle \pm \frac{1}{\sqrt{2}}|zx\rangle$, and $|3\rangle =\left|x^{2}-y^{2}\right|$ where the “+” and “−” signs stand for Cr1 and Cr2, respectively [13]. Sometimes these orbitals are denoted as |xy〉, |yz−zx〉, and |yz+zx〉 in the local coordinate frame, respectively [45]. The relevant orbital-projected DOS of Cr1 and Cr2 in Cr(NCN)_2_ and CrO_2_ from GGA+*U* calculations given in Figure 4 are performed on symmetry grounds; we projected densities in majority spin channel of the |1>, |2>, and |3> symmetry with respect to the crystal coordinate frame. We also inserted the numerical orbital populations obtained by integration up to the Fermi energy in Figure 4. In Cr(NCN)_2_, the projected DOS of |1>, |2>, and |3> of Cr1 and Cr2 are similar, and the orbital populations are quite close, reflecting almost the same spin moment of Cr1 and Cr2 discussed before. In CrO_2_, the projected DOS |1> and |3> of both Cr ions are similar with the close orbital population. However, the projected DOS of |2> of Cr1 and Cr2 are dissimilar, with the difference of 0.226 in the orbital population. Obviously, the different spin moments of Cr1 and Cr2 in CrO_2_ mainly result from |2>.

For both compounds, |1> mainly locates below −1.0 eV and is localized with an energy gap of 1.9 eV for Cr(NCN)_2_ and 1.3 eV for CrO_2_ at the Fermi level. Moreover |1> is less hybridized with O 2*p* orbitals (Figure 3), suggesting a possible direct Cr–Cr interaction. |1> of Cr ions in Cr(NCN)_2_ have a smaller orbital occupation (1.089 and 0.979) than those in CrO_2_ (1.349 and 1.376). In both compounds, the electrons in |2> and |3> are itinerantly crossing the Fermi level with a strong hybridization with N/O 2*p* orbitals (Figure 3). The Fermi level is mainly occupied by |3> in Cr(NCN)_2_, while that of CrO_2_ is occupied by both |2> and |3>. Besides, |3> in Cr(NCN)_2_ (0.66 and 0.72) has a bigger orbital population than that in CrO_2_ (0.53 and 0.50). The total orbital populations of 3*d* orbitals of Cr1 and Cr2 are 2.985 and 2.995 in Cr(NCN)_2_, and 3.289 and 3.069 in CrO_2_, respectively, larger than 2.0 of ideal Cr^4+^ ion (3*d*^2^). That probably implies a large negative charge transfer from N/O 2*p* orbitals to Cr 3*d* orbitals. In short, the different spin moments of Cr1 and Cr2 in CrO_2_ mainly result from |2>. The conductivity of Cr(NCN)_2_ mainly results from |3>, however, that of CrO_2_ is attributed from both |2> and |3>.

In order to gain insight into the magnetic coupling between the Cr1 and Cr2 ions, we analyze the three-dimensional electronic-density contour plots near the Fermi level, according to orbital-projected DOS. Figure 4c,d show the density of electron distribution in the energy regions of (–2.20)–(–1.60) eV for Cr(NCN)_2_ and (–1.57)–(–1.35) eV for CrO_2_ (purple arrows in Figure 3 and in bottom panel of Figure 4b). In this region, for both compounds, the electrons are almost occupied in Cr atoms |1> orbitals, forming *d*–*d* direct superexchange in the edge-shared CrN_6_/CrO_6_ chain. This superexchange, where an electron is assumed to drift from one cation, exists in the compounds with edge-sharing or face-sharing octahedral chain or dimer. The *d*–*d* hopping is very important for early 3*d* meals such as Ti, V, and Cr [46]. As shown in Figure 4a,b, Cr^4+^ ion in both Cr(NCN)_2_ and CrO_2_ has |1> orbital only partially occupied for the up-spin state, that is, the integrated value in the full energy range is around 2, while the orbital occupation (integration below Fermi level) is only around 1, thus an intra-chain ferromagnetic interaction is expected [46,47]. The calculated effective exchange coupling constants of intra-chain *J*_1_ from GGA+*U* (Table 1) are 20.5 and 12.1 meV for Cr(NCN)_2_ and CrO_2_, respectively, indicating a stronger intra-chain coupling in Cr(NCN)_2_. Figure 4e,f shows the density of electron distribution in the energy regions of (–−0.69)–− (–0.40) eV and (–0.64)–(–0.37) eV for Cr(NCN)_2_ and CrO_2_, respectively (green arrows in Figure 3 and middle panel of Figure 4a,b). In this region, the electrons are mainly occupied by Cr |2> orbitals and O 2*p* orbitals, forming *dpπ*–*dpπ* correlation ferromagnetic superexchange between Cr1 and Cr2 sites. In CrO_2_, this interaction is strong, while it is cut off by C atoms in Cr(NCN)_2_. This is consistent with the results of the inter-chain coupling constants *J*_2_ from GGA+*U* calculations, that is, 21.5 meV for Cr(NCN)_2_ and 31.2 meV for CrO_2_. Figure 4g,f show the density of electron distribution in the energy regions near the Fermi level, that is, from −0.10–0.0 eV for Cr(NCN)_2_ and −0.10–0.0 eV for CrO_2_, respectively (blue arrows in Figure 3 and bottom panel of Figure 4a,b). In this region, the electrons are mainly occupied by Cr |3> orbitals and O 2*p* orbitals, also forming *dpπ*–*dpπ* correlation ferromagnetic superexchange between Cr1 and Cr2 sites. However, the electrons are more localized in Cr(NCN)_2_ than those in CrO_2_ because of the large size ligand of NCN^2−^. Obviously, there are two magnetic couplings, that is, direct *d*–*d* exchange and indirect *dpπ*–*dpπ* superexchange, dominating the ferromagnetic properties of Cr(NCN)_2_ and CrO_2_. In Cr(NCN)_2_, the strengths of the intra- and inter-chain couplings are comparative, however, in CrO_2_, there is a large difference between them, that is, 12.1 meV for intra-chain and 31.2 meV for inter-chain.

## 4. Conclusions

In summary, on the basis of the results of density functional theory with explicit electronic correlation, Cr(NCN)_2_ is a ferromagnetic and half-metal material, and stable both thermodynamically and elastically. It was predicted to be a tetragonal structure in the space group of *P*4_2_/*mnm* with an asymmetrical [N–C≡N]^2−^ ligand; as a result, it would be the first transition-metal cyanamide with partially filled 3*d* orbitals. A comparative study on the electronic structures of Cr(NCN)_2_ and CrO_2_ presents that the Cr atoms in both compounds are in one crystallographically equivalent site, however, in CrO_2_, the Cr atoms at the corner and center sites have different magnetic moments and orbital occupancies, and there is a large difference between the intra- (Cr atoms at the same site) and inter-chain (Cr atoms at the different sites) magnetic couplings. This difference is significantly weakened by C atoms in Cr(NCN)_2_. Thus, ferromagnetically half-metallic Cr(NCN)_2_ might be an interesting spintronic material, and we hope it could be synthesized in the future.

## Figures and Tables

**Table 1:** Total energies (*E*_total_) (m*e*V) of the ferromagnetic (FM), anti-ferromagnetic (AFM)1, and AFM2 states per formula unit relative to that of the FM state for Cr(NCN)_2_ and CrO_2_; the effective exchange coupling constants (*C*) (m*e*V); the calculated saturated magnetic moment (*M*) of Cr, N, and O ions; and the total magnetic moment per formula unit (*μ*_B_), as coming from the GGA and density functional theory with explicit electronic correlation (GGA+*U*) (*U* = 3 *e*V) calculations.

		Cr(NCN)_2_		CrO_2_	
		GGA	GGA+*U*	GGA	GGA+*U*
***E*_total_ (meV)**	**FM**	0	0	0	0
	**AFM1**	109.1	172.3	159.6	249.5
	**AFM2**	57.9	127.2	115.1	148.9
***C* (meV)**	***J*_1_**	1.7	20.5	17.7	12.1
	***J*_2_**	13.7	21.5	19.9	31.2
***M* (*μ*_B_)**	***M*_Cr_**	2.18	2.63	2.11	2.38
		2.18	2.62	2.05	2.31
	***M*_N/O_**	−0.06	−0.11	−0.05	−0.13
		−0.06	−0.10	−0.06	−0.12
		−0.06	−0.12		
		−0.06	−0.11		
	***M*_total_**	2.01	2.26	1.97	2.09

## Supplementary Materials

The following are available online at https://www.mdpi.com/1996-1944/13/8/1805/s1. Table S1. The structure parameters of predicted Cr(NCN)_2_. Thermodynamically stability. Figure S1. Ordered spin arrangements of Cr(NCN)_2_ and CrO_2_ designed as (a) FM, (b) AFM1, and (c) AFM2 employed to extract the spin exchange parameters *J*_1_ and *J*_2_. Figure S2. Band structures near the Fermi energy for (a) Cr(NCN)_2_ and (b) CrO_2_ from GGA+*U* (*U* = 3 *e*V) calculations.
