# Supplementary material for: Ferromagnetic Half-Metal Cyanamides Cr(NCN)2 Predicted from First Principles Investigation
Source: Materials (Basel). 2020 Apr 11;13(8):1805. doi: 10.3390/ma13081805 (PMC7216073; doi:10.3390/ma13081805)
Supplement: Supplementary file 1 [file materials-13-01805-s001.pdf]

materials

Supplementary Materials

Ferromagnetic Half-Metal Cyanamides $Cr(NCN)_2$ Predicted from First Principles Investigation

Zhilue Wang [1], Shoujiang Qu [1], Hongping Xiang [1,*], Zhangzhen He [2,*], and Jun Shen [1]

1 School of Materials Science and Engineering, Tongji University, 4800 Caoan Road, Shanghai 201804, China; wangzl@tongji.edu.cn (Z.W.); qushoujiang@tongji.edu.cn (S.Q.); junshen@tongji.edu.cn (J.S.)
2 State Key Laboratory of Structural Chemistry, Fujian Institute of Research on the Structure of Matter, Chinese Academy of Sciences, Fuzhou, Fujian 350002, China
* Correspondence: xianghp@tongji.edu.cn (H.X.); hezz@fjirsm.ac.cn (Z.H.)

Received: 2 March 2020; Accepted: 9 April 2020; Published: 11 April 2020

Table S1. The structure parameters of predicted $Cr(NCN)_2$.

	**$Cr(NCN)_2$**
Crystal system	*Tetrahedral*
Space group	*$P4_2/mnm$*
a	8.04 Å
b	8.04 Å
c	3.13 Å
α	90°
β	90°
γ	90°
V	201.94 $Å^3$
Z	2
Cr 2a	(0 0 0)
C 4g	(0.2238 0.2238 0)
N1 4g	(0.1126 0.1126 0)
N2 4g	(0.3299 0.3299 0)
C-N1	1.264 Å
C-N2	1.206 Å
Cr-N1	2.032 Å
Cr-N2	1.934 Å
∠N-C-N	180°

Thermodynamically stability

The possible reaction routes were summarized in Equation (S1) and Equation (S2), based on successfully synthesized compounds Cr_2NCN_3 and MNCN (M = Mn, Fe, Co, Ni). For the reactants, the space groups of $CrCl_3$, Cl_2(solid molecular), Li_2NCN, and LiCl are *C*12/*m* [1], *Cmca* [2], *I*4/*mmm* [3], and *Fm*-3*m* [4], respectively.

$$2CrCl_3 + Cl_2 + 4Li_2(NCN) = 2Cr(NCN)_2 + 8LiCl \tag{1}$$

$$2CrCl_3 + Cl_2 + 4Zn(NCN) = 2Cr(NCN)_2 + 4ZnCl_2 \tag{2}$$

$$Cr + 2C + 2N_2 = Cr(NCN)_2 \tag{3}$$

The calculated chemical reaction energies are -7.46 eV in Equation (S1) and -4.74 eV Equation (S2) for $Cr(NCN)_2$. The negative value indicates that the designed reactions are attractive candidates. The formation enthalpy calculated from the direct route (Equation (S3)) starting with elements Cr

(I*m*-3*m*), C (*P*6$_3$/*mmc*) and N_2 (*P*6$_3$/*mmc*), is −13.54 eV, indicating that Cr(NCN)$_2$ is thermodynamically stable with respect to the elements.

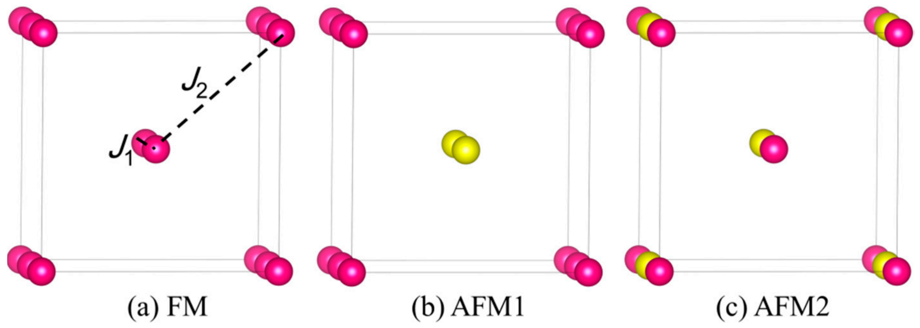

Figure S1. Ordered spin arrangements of Cr(NCN)$_2$ and CrO_2 designed as (**a**) FM, (**b**) AFM1, and (**c**) AFM2 employed to extract the spin exchange parameters J_1 and J_2. Pink and yellow spheres indicate different orientations of the spin moments residing on the transition-metal M atoms.

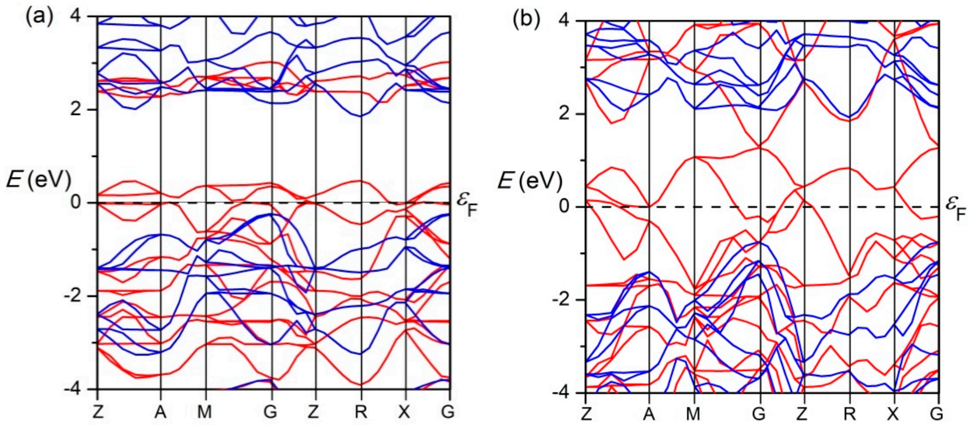

Figure S2. Band structures near the Fermi energy for (**a**) Cr(NCN)$_2$ and (**b**) CrO_2 from GGA+*U* (*U* = 3 *e*V) calculations.

References

1. Morosin, B.; Narath, A. X-Ray Diffraction and nuclear quadrupole resonance studies of chromium trichloride. *J. Chem. Phys.* **1964**, *40*, 1958, doi: 10.1063/1.1725428.
2. Collin, R.L. The crystal structure of solid chlorine. *Acta Crystallogr.* **1956**, *9*, 537–537, doi: 10.1107/S0365110X56001467.
3. Down, M.G.; Haley, M.; Hubberstey, P.; Pulham, R.J.; Thunder, A.E. Solutions of lithium salts in liquid lithium: preparation and X-ray crystal structure of the dilithium salt of carbodi-imide (cyanamide). *J. Chem. Soc., Dalton Trans.* **1978**, *9*, 1407–1411, doi: 10.1039/dt9780001407.
4. Pietro, C. Direct determination of self-consistent total energies and charge densities of solids: A study of the cohesive properties of the alkali halides. *Phy. Rev. B* **1992**, *46*, 2008–2014, doi: 10.1103/PhysRevB.46.2008.

© 2020 by the authors. Submitted for possible open access publication under the terms and conditions of the Creative Commons Attribution (CC BY) license (http://creativecommons.org/licenses/by/4.0/).
